# Electron collimator in Weyl semimetals with periodic magnetic barriers

**DOI:** 10.1038/s41598-019-47334-x

**Published:** 2019-07-29

**Authors:** Xunwu Hu, Fang Cheng

**Affiliations:** Department of Physics and Electronic Science, Changsha University of Science and Technology, Changsha, 410004 China

**Keywords:** Spintronics, Topological insulators

## Abstract

We investigate theoretically the effect of periodic magnetic barriers on the transport for a Weyl semimetal. We find that there are momentum and spin filtering tunneling behaviors, which is controlled by the numbers of the magnetic barriers. For the tunneling through periodic square-shaped magnetic barriers, the transmission is angular *φ* asymmetry, and the asymmetrical transmission probability becomes more pronounced with increasing the superlattice number *n*. However, the transmission is symmetric with respect to angle *γ*, and the window of the transmission become more and more narrower with increasing the number of barriers, i.e., the collimator behavior. This feature comes from the electron Fabry-Pérot modes among the barriers. We find that the constructive interference of the backscattering amplitudes suppress transmissions, and consequently form the minigaps of the transmission. The transmission can be switched on/off by tuning the incident energies and angles, the heights and numbers of the magnetic barriers, and result in the interesting collimator behavior.

## Introduction

In recent years, Weyl semimetal, has attracted extensive interest in the physics community due to its novel physical properties and potential applications^1–6^. The energy dispersion of quasi-particles in Weyl semimetal support nodal points that result from splitting of Dirac nodes^7,8^. Weyl cones and surface Fermi arcs have been observed by the angle-resolved photoemission spectroscopy in Weyl semimetals, such as in TaAs^9,10^, NbAs^11^ and TaS^12^. There are exotic properties in Weyl semimetals, such as the chiral anomaly^13–15^, a negative magnetoresistance^16^, the Hall effect^17^ and other anomalous transport properties^18,19^. Moreover, the electrons in Weyl semimetals have high mobility and chirality, therefore they have excellent application prospects in transport^20–25^.

Weyl semimetals, the three dimensional (3D) analogue of graphene, have linear dispersions around the Weyl points. It is convenient to operate the Dirac fermions in graphene by means of the external electric field and magnetic field^26–31^. Experimentally applying bias to the gate region or alkali doping can adjust the Fermi level in 3D Dirac semimetals^32,33^. Recently, a significant amount of attention was devoted to magnetotransport, which was studied theoretically and experimentally^34–36^.

Electron tunnelling through double magnetic barriers on the surface of a topological insulator was studied^37^. Subsequently, the effect of the periodic barriers on the surface of HgTe was studied^38^. After the realization of Weyl semimetals, the electron tunnelling through double magnetic barriers on the surface of a Weyl semimetal was investigated^39^. In this paper, we study theoretically the effect of periodic magnetic barriers on the Weyl semimetal surface. There are momentum and spin filtering behaviors, which is tuned by the numbers of the magnetic barriers. It is interesting to notice that electron collimator behavior can be found due to the electron Fabry-Pérot modes induced by the multiple reflections among barriers, the interference of the backscattering processes leads to minigap opening of the transmissions, i.e., the complete suppression of the transmission.

## Methods

**Figure 1:**
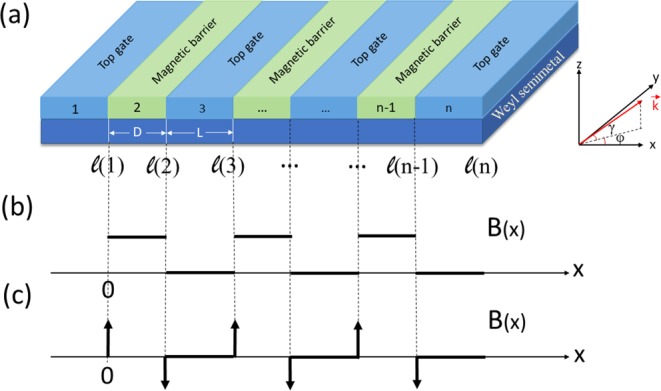
(**a**) The Weyl semimetal system with periodic magnetic barriers. The red arrow is incident wavevector with incident angles *γ* and *φ*. (**b**) The magnetic field *B*(*x*) of square-shaped barriers. (**c**) The magnetic field *B*(*x*) of delta-function-shaped barriers.

We consider *n* period magnetic barriers with the same width *D* (see Fig. 1). The distance between the neighbouring barriers is *L*. The low energy Hamiltonian of the Weyl fermion under the magnetic field is

1 $$H={v}_{F}(\sigma \cdot ({\bf{p}}+e{\bf{A}})),$$

where *v*_*F*_ is the Fermi velocity, and *σ* is Pauli matrices, the vector potential generated by the magnetic field **B**(*x*) = (0, 0, *B*) is **A** = (0, *A*_*y*_, 0). Here we have neglected the Zeeman splitting because of very small band shift^40^. The electrical potential can make electron’s wave-vector in a Weyl semimetal with tilted energy dispersion shift due to broken Lorentz symmetry^22–24^. It is possible to using the dispersion tilt instead of magnetic field barriers to obtain the electron collimation behavior. The Hamiltonian looks very similar with that of graphene and the surface states of 3D topological insulators (TIs), the tunneling processes were studied theoretically before^37^. The dominant difference between the Weyl semimetal and graphene and 3D TIs are that the Dirac cone in Weyl semimetal is a 3D system, while the others are two-dimensional systems. For convenience, we use dimensionless units: *l*_*B*_ = [ℏ/*eB*_0_]^1/2^, *E*_0_ = ℏ*v*_*F*_/*l*_*B*_, **r** → *l*_*B*_**r**, **k** → **k**/*l*_*B*_, **B**(*x*) → *B*_0_**B**(*x*), *E* → *E*_0_*E*, the Hamiltonian becomes

2 $$H=(\begin{array}{cl}{k}_{z} & {k}_{x}-i({k}_{y}+{A}_{y})\\ {k}_{x}+i({k}_{y}+{A}_{y}) & -{k}_{z}\end{array}),$$

where the three components of wavevector can be written *k*_*x*_ = *k*_*F*_cos*γ*cos*φ*, *k*_*y*_ = *k*_*F*_cos*γ*sin*φ*, *k*_*z*_ = *k*_*F*_sin*γ* with the Fermi wavevector ${k}_{F}^{2}={k}_{x}^{2}+{k}_{y}^{2}+{k}_{z}^{2}$. By solving eq. [2], we obtain the relationship: ${E}^{2}-{k}_{z}^{2}={k}_{x}^{2}+{({k}_{y}+{A}_{y})}^{2}$.

There are two different magnetic field profiles, i.e., square-shaped and delta-function-shaped magnetic fields. Depositing superconducting strips above the Weyl semimetal with a magnetic field, we can obtain the square-shaped magnetic fields^41^. The magnetic barrier strength is beyond 30 T^42^. The vector potential of a square-shaped magnetic barrier is *A*_*y*_(*n*) = *B*(*x* − *L*(*n* − 2)/2) for *mod*(*n*, 2) = 0, and *A*_*y*_(*n*) = (*n* − 1)*BD*/2 for other cases. Depositing ferromagnetic metallic strips on Weyl semimetal through a thin oxide layer, we can obtain the delta-function-shaped magnetic field^43,44^. And the magnetic field strength is achieved experimentally around 3.75 T^45^. The vector potential of a delta-shaped magnetic barrier reads *A*_*y*_(*n*) = *BD* for *mod*(*n*, 2) = 0, and *A*_*y*_(*n*) = 0 for other cases.

The momentum *p*_*y*_ and *p*_*z*_ along the interface are good quantum numbers because of the translational invariance. The wave function reads ${\rm{\Phi }}(\overrightarrow{r})={\rm{\Psi }}(x)\exp (i{k}_{y}y+i{k}_{z}z)$, where *k*_*y*_ and *k*_*z*_ are the wave numbers. Assuming a Weyl fermion incident from the left electrode, the wave functions in the left and right regions can read Ψ_*L*_ = *ψ*^+^ + *rψ*^−^,Ψ_*R*_ = *tψ*^+^, where ${\psi }^{+}=\frac{({k}_{x}(x)+i({k}_{y}+{A}_{y})){e}^{i{k}_{x}(x)x}}{E+{k}_{z}}$, ${\psi }^{-}=\frac{(-{k}_{x}(x)+i({k}_{y}+{A}_{y})){e}^{-i{k}_{x}(x)x}}{E+{k}_{z}}$, in which *r* and *t* are the reflections and transmission coefficients. Applying the continuity of the wave functions at the boundaries, we obtain

3 $$(\begin{array}{l}1\\ r\end{array})={(\begin{array}{ll}1 & 1\\ \frac{{k}_{x}(1)+i{k}_{y}(1)}{E+{k}_{z}} & \frac{-{k}_{x}(1)+i{k}_{y}(1)}{E+{k}_{z}}\end{array})}^{-1}\sum _{i=1}^{n-2}S(l{(i)}^{+}){[S(l{(i+1)}^{-})]}^{-1}(\begin{array}{l}1\\ \frac{{k}_{x}(n)+i{k}_{y}(n)}{E+{k}_{z}}\end{array})t{e}^{i{k}_{x}(n)l(n)},$$

where *l*(*i*) is the position of the interface, *l*(*i*)^±^ = *l*(*i*) ± *δ* with infinitesimal positive *η*, *S*(*l*(*i*)^+^) and *S*(*l*(*i* + 1)^−^) can be given by the *x*-dependent 2 × 2 matrixes, whose columns are constructed by the independent eigenstates of the Eq. (2). The transfer matrix of square-shaped magnetic barrier case can be expressed in general form as

4 $$\{\begin{array}{l}S(x)=(\begin{array}{ll}{D}_{\frac{v}{2}-1}(\sqrt{2}({k}_{y}+{A}_{y})) & {D}_{\frac{v}{2}-1}(-\sqrt{2}({k}_{y}+{A}_{y}))\\ i\sqrt{\frac{2}{v}}{D}_{\frac{v}{2}}(\sqrt{2}({k}_{y}+{A}_{y})) & -i\sqrt{\frac{2}{v}}{D}_{\frac{v}{2}}(-\sqrt{2}({k}_{y}+{A}_{y}))\end{array}),mod(n,2)=0,\\ S(x)=(\begin{array}{cc}{e}^{i{k}_{x}(x)x} & {e}^{-i{k}_{x}(x)x}\\ \frac{({k}_{x}(x)+i({k}_{y}+{A}_{y})){e}^{i{k}_{x}(x)x}}{E+{k}_{z}} & \frac{(-{k}_{x}(x)+i({k}_{y}+{A}_{y})){e}^{-i{k}_{x}(x)x}}{E+{k}_{z}}\end{array}),{\rm{otherwise}}.\end{array}$$

Here *v* = ${E}^{2}-{k}_{z}^{2}$. Note that the wave function forms of both square-shaped and delta-function-shaped magnetic field profiles are same at *mod*(*n*, 2) ≠ 0, but the vector potential *A*_*y*_ is different for the two different magnetic field profiles. The magnetic unit is *B*_0_ = 1*T*, the energy unit is *E*_0_ = 26 *meV*, and the length unit is *l*_*B*_ = 26 *nm* in the following calculation. We can obtain the transmission probability using the scattering-matrix technique.

## Results and Discussions

### Transport with periodic square-shaped magnetic barriers

**Figure 2:**
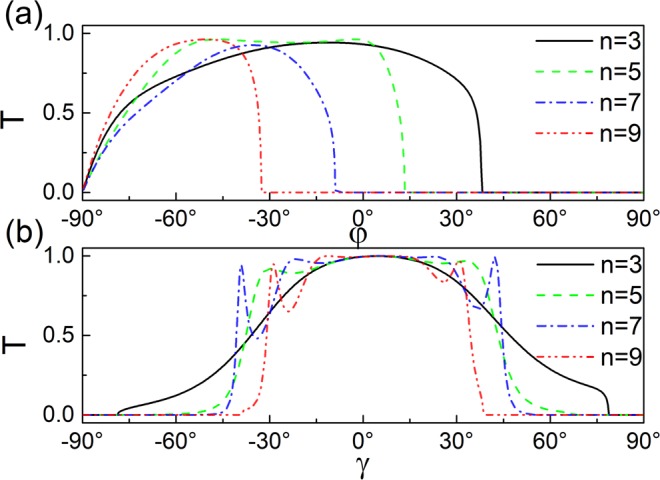
Transmission probability in the case of periodic square-shaped magnetic barrier versus the incident angles (**a**) *φ* with *γ* = *π*/6, (**b**) *γ* with *φ* = −*π*/4. The incident energy is *E*_*F*_ = 78 *meV*, the barrier width is *D* = 26 *nm*, distance is *L* = 26 *nm* and magnetic field is *B* = 1 *T*.

The transmission probability for periodic square-shaped magnetic barriers structure under successively increasing number of modulations *n* = 3, 5, 7, 9 are plotted as a function of the incident angle in Fig. 2. As shown in Fig. 2(a), the transmission is angular *φ* asymmetric due to the inhomogeneous magnetic field. The asymmetrical transmission probability become more distinct with increase of the superlattice number *n*, which means that a wave-vector filtering is more astonishing for bigger superlattice number. The boundary of *T* = 0 can be obtained by the equation *E*_*F*_*B*_0_*l*_*B*_ cos*γ*(1 − sin*φ*) = (*n* − 1)*BDE*_0_/2. The transmission falls sharply and even to zero when the incident angle *φ* is past a critical value *φ*_*c*_ = arcsin(1 − (*n* − 1)*BDE*_0_/2*E*_*F*_*B*_0_*l*_*B*_cos*γ*). The wavevector becomes imaginary, evanescent modes in the outgoing region appear, thus the transport is switched off. The transmission is symmetrical to the incident angle *γ* (see Fig. 2(b)). The transport is concentrated in a narrower area with the increase superlattice number *n*, according to the relation ${k}_{x}{l}_{B}=\sqrt{{(\frac{{E}_{F}\cos \gamma }{{E}_{0}})}^{2}-{(\frac{{E}_{F}\cos \gamma \sin \phi }{{E}_{0}}+\frac{(n-1)BD}{2{B}_{0}{l}_{B}})}^{2}}$. For a fixed incident energy, the transmission falls sharply and even to zero when *γ* is past a critical value *γ*_*c*_ = arccos(*n* − 1)*BDE*_0_/2*E*_*F*_*B*_0_*l*_*B*_(1 − sin*φ*).

**Figure 3:**
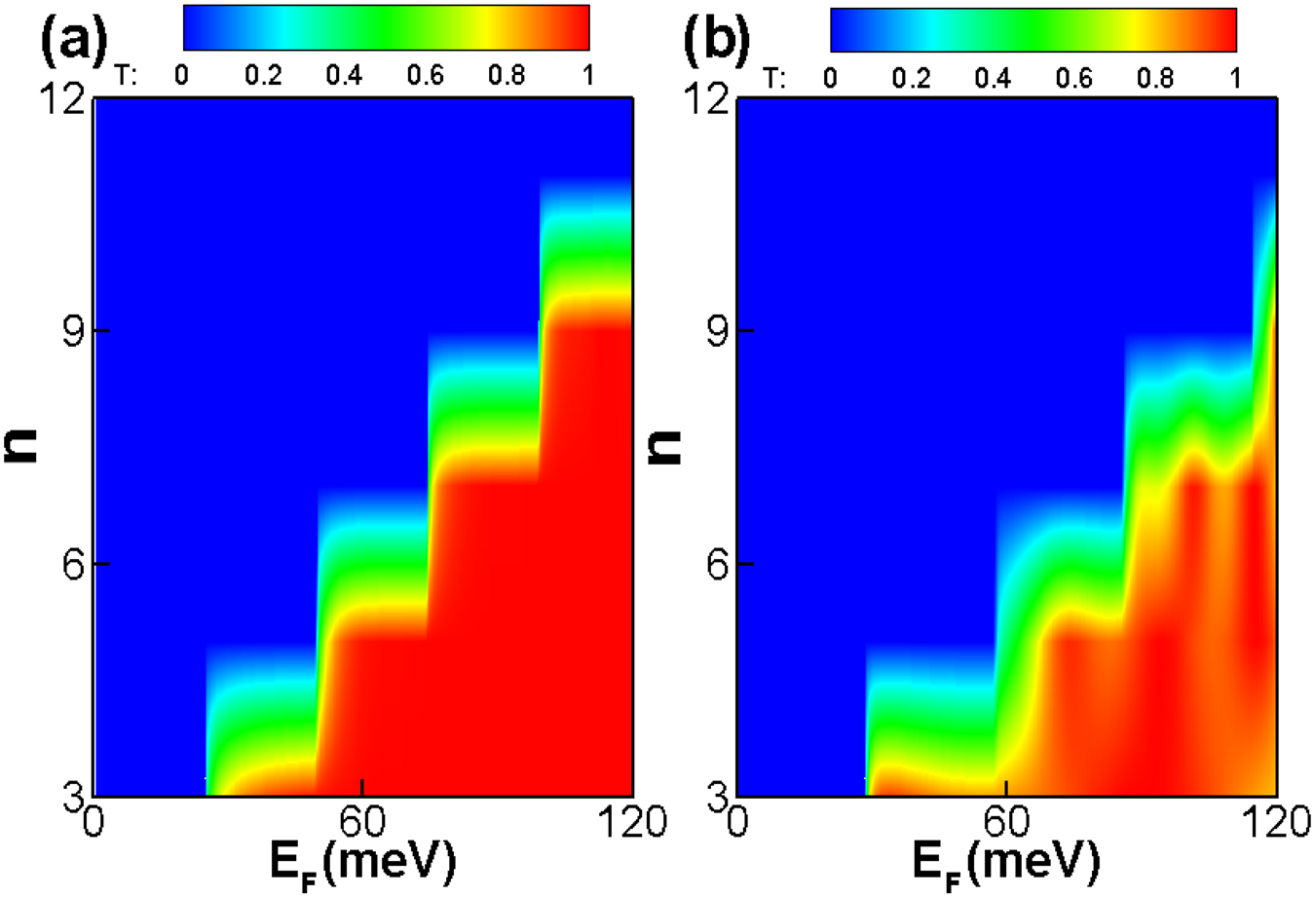
The (*E*_*F*_, *n*) dependence of tunneling probability through square-shaped periodic magnetic barrier for (**a**) *γ* = 0 and (**b**) *γ* = *π*/6. The incident angle is *φ* = 0, *D* = 26 *nm*, *L* = 26 *nm* and *B* = 1 *T*.

It is interesting to see the effect of the incident energy and superlattice number *n* on the perfect transmission. Figure 3 is the contour plot of the transmission probability *T*(*E*_*F*_, *n*) for a periodic square-shaped magnetic barriers structure. The incident angle is *γ* = 0 for Fig. 3(a) and *γ* = *π*/6 for Fig. 3(b), respectively. The tunneling is switched on when the incident energy *E*_*F*_ is past the critical value, and the critical incident energy becomes bigger with increasing superlattice number *n*. The boundary of the total reflection region is determined by the relation *E*_*F*_ ≤ (*n* − 1)*BDE*_0_/2*B*_0_*l*_*B*_cos*γ*(1 − sin*φ*). In the case of *γ* = 0 as shown in Fig. 3(a), there is not oscillating behavior due to without quasibound states. However, for *γ* = *π*/6 as shown in Fig. 3(b), there is the reflection in the *z* direction, therefore there is oscillating behavior of the transmission probability because of quasibound states. This is accordance with our previous work^39^. Reference^39^ has investigated the transport in a magnetic/normal/magetic hybrid structure on the surface of a Weyl semimetal. This present work focuses on the influence of the number of superlattice layers on transport.

**Figure 4:**
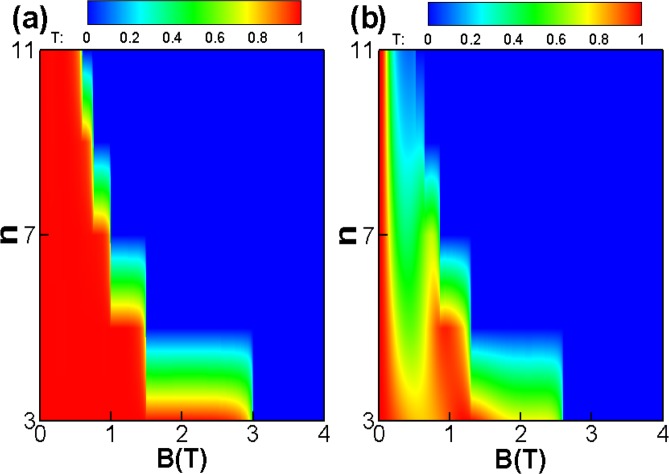
The (*B*, *n*) dependence of transmission probability through square-shaped periodic magnetic barrier for the incident angle for (**a**) *γ* = 0 and (**b**) *γ* = *π*/6. The incident angle *φ* = 0, *E*_*F*_ = 78 *meV*, *D* = 26 *nm*, and *L* = 26 *nm*.

Figure 4 is the contour plot of the transmission probability *T*(*B*, *n*) through a periodic square-function-shaped magnetic barriers, in the case of incident energy *E*_*F*_ = 78 *meV*, incident angle *φ* = 0, the width *D* = 26 *nm*, distance *L* = 26 *nm*. The incident angle is *γ* = 0 for Fig. 4(a) and *γ* = *π*/6 for Fig. 4(b), respectively. One can see clearly that the tunneling is totally forbidden at the cut-off magnetic field *B*, and the critical magnetic field becomes weaker as increasing superlattice number *n*. The total reflection region is dertermined by *B* ≥ (*n* − 1)*E*_*F*_*l*_*B*_ cos*γ*(1 − sin*φ*)*B*_0_/2*DE*_0_. The magnetic field *B* strongly affect and control the transmission. As we known, the cyclotron orbit radius is *R* = *vm*/*qB*. When *B* exceeds a certain magnetic field value, then the cyclotron orbit radius *R* is less than the width *D*, so the incident electron will back out of the barrier region. Therefore, there is a sharp transition where the transmission probability *T* becomes almost zero beyond a certain magnetic field value. For *γ* = *π*/6 as shown in Fig. 4(b), there is quasibound states due to the reflection in the *z* direction, therefore there is oscillating behavior of the transmission probability, which is consistent with Fig. 3(b).

**Figure 5:**
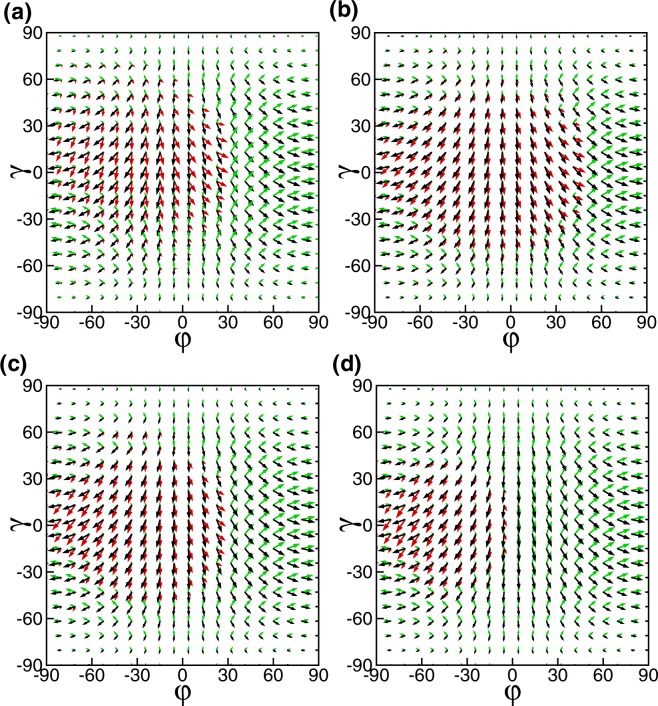
The angular dependence of the spin orientations. The black arrows respond to the incident electrons, the red arrows to the transmitted electrons, and the green arrows to the reflected electrons, respectively. The barrier width *D* = 26 *nm*, *L* = 78 *nm*, and *B* = 1. The superlattice *n* = 3 and the incident energy is (**a**) *E*_*F*_ = 52 *meV*, (**b**) *E*_*F*_ = 104 *meV*, respectively. The incident energy is *E*_*F*_ = 104 *meV* and the superlattice is (**c**) *n* = 5, (**d**) *n* = 9, respectively.

Most interestingly, we can control spin transport on Weyl semimetal. The spin orientation is plotted as function of *φ* and *γ* for a parallel configuration square-shaped magnetic barriers at different incident energy *E*_*F*_ = 52 *meV* and 104 *meV*, respectively (Fig. 5(a,b)). There is a rotation angle between the transmitted electron spin and the incident electron spin. And the rotation angle depends on the electron energy and the vector potential *A*_*y*_, as shown in Fig. 5(a,b). The spin orientation is plotted as function of *φ* and *γ* for square-shaped magnetic barriers with a fixed incident energy *E*_*F*_ = 104 *meV* for different superlattice *n* = 5 and *n* = 9, respectively (Fig. 5(c,d)). The *A*_*y*_ in the outgoing region depends on the magnetic barrier height and width, therefore the spin and direction of motion of the transmitted electrons can be controlled by the number of the barriers. The decrease of incident energy and/or the increase of magnetic barrier number *n* can inhibit transmission probability.

### Transport in the presence of periodic delta-function-shaped barriers

**Figure 6:**
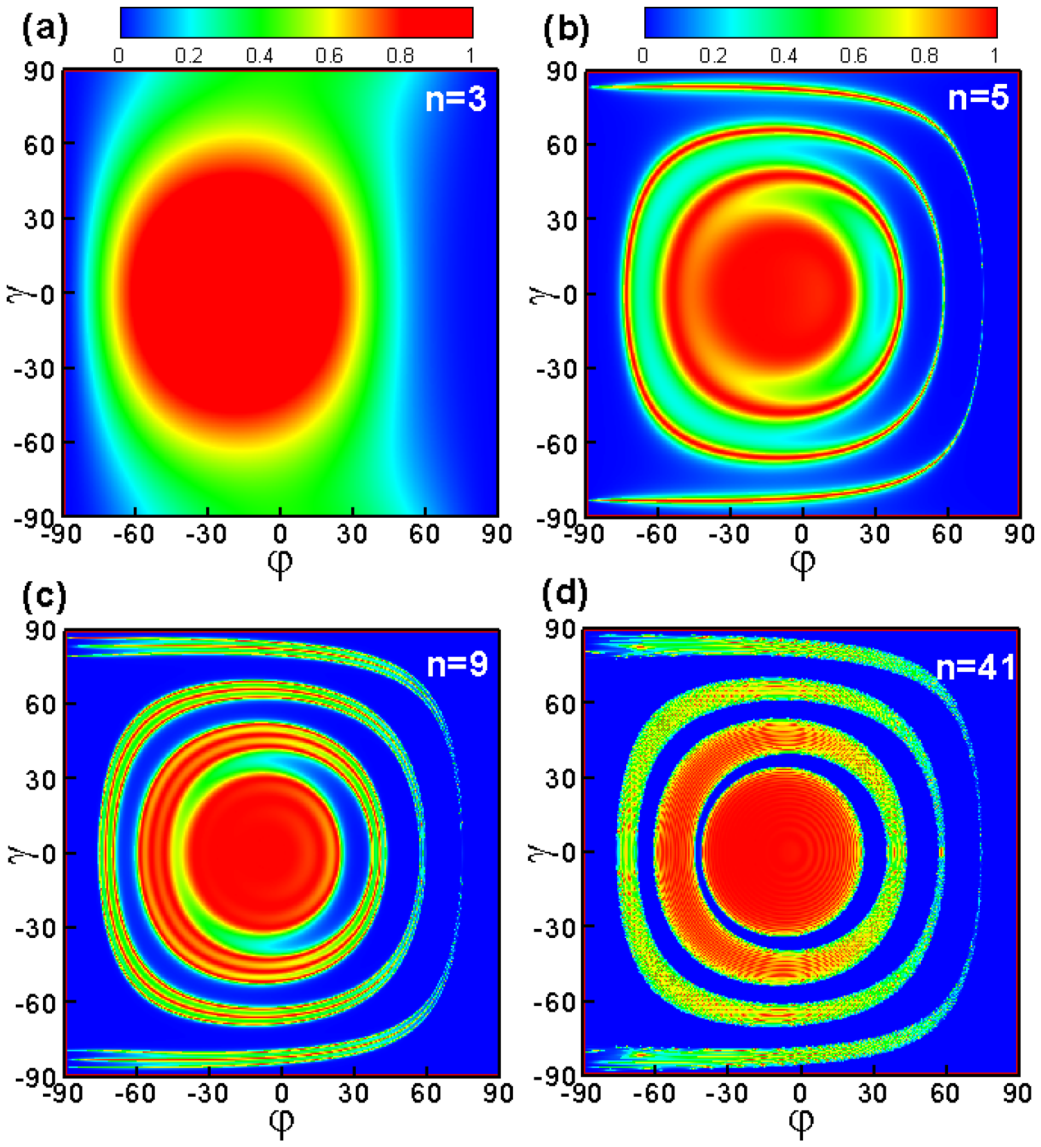
The angular dependence of the transmission probability through delta-function-shaped periodic magnetic barrier. *E*_*F*_ = 78 *meV*, *D* = 26 *nm*, *L* = 26 *nm*, and *B* = 1*T*.

In the following we consider tunneling in the presence of the periodic delta-function-shaped magnetic barriers under successively increasing number of modulations *n* = 3, 5, 9, 41. Figure 6 is contour plot of transmission probability *T*(*φ*, *γ*). Here we have fixed that *E*_*F*_ = 78 *meV*, *B* = 1 *T*, *D* = 26 *nm*, *L* = 26 *nm*. For the case of a single barrier structure *n* = 3 (Fig. 6(a)), there is no oscillating behavior, and there is a high transmission probability in the range of *γ* = [−60°, 60°] and *φ* = [−60°, 30°]. For the case of a double barrier structure *n* = 5 (Fig. 6(b)), the transmission becomes very different from that of the single barrier structure. There are Fabry-Pérot modes due to the multiple reflections in both *y* and *z* directions. And the transmission for the delta-function-shaped magnetic barrier case has obvious resonant behavior in the case of large positive incident angles. On the contrast, tunneling for the square-shaped magnetic barrier case is completely forbidden for large positive angle *φ* [see Fig. 2(a)] and large angle |*γ*| [see Fig. 2(b)]. The interference behavior becomes more obvious with increasing *n*. (see Fig. 6(c,d)). Except the Fabry-Pérot modes between the two barriers, there is interference of the backscattering by different layers when *n* ≥ 7. It is interestingly to see that the boundary of perfect transmission and totally forbidden in the central region is more clearly at *n* = 41, which is as shown in Fig. 6(d). This is because the number of layers *n* increases, the interference effect becomes more obvious. There is constructive inteference when *k*_*x*_ is an integer multiple of *π*/(*L* + *D*)^38^.

**Figure 7:**
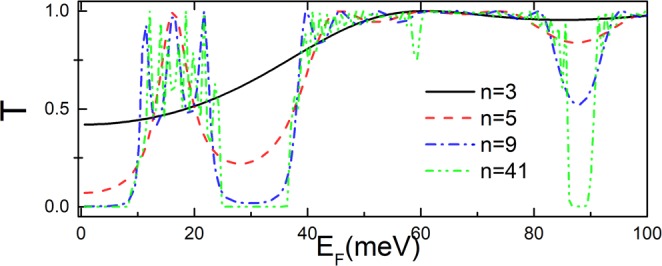
Transmission probability through the delta-function-shaped periodic magnetic barrier versus incident energy *E*_*F*_. The incident angles *γ* = *π*/6, *φ* = 0, the barrier width *D* = 26 *nm*, distance *L* = 26 *nm* and magnetic field *B* = 1*T*.

Figure 7 shows the tunneling in the case of the periodic delta-function-shaped magnetic barriers as a function of Fermi energy under successively increasing number of modulations *n* = 3, 5, 9, 41. For a single barrier structure *n* = 3 (black solid line in Fig. 7), there is no oscillating behavior. For a double barrier structure *n* = 5 (red dashed line in Fig. 7), we find a obvious oscillating behavior stems from Fabry-Pérot modes between the two barriers. The first transmission peak appears nearby *E*_*F*_ ≈ 18 *meV*, and the transmission valley appears nearby *E*_*F*_ ≈ 30 *meV*. With increasing *n*, there is a huge oscillation in the transmission peak nearby *E*_*F*_ ≈ 18 *meV*, this is because the interference of the backscattering by different layers. When the number *n* of modulation is further increased, it is interesting to see that the transmission pronounced dip near *E*_*F*_ ≈ 88 *meV*, and a totally forbidden near *E*_*F*_ ≈ 25 *meV*, 38 *meV*]. The increasingly strong interferences of backscattering cause superlattice minigaps, thus completely suppress the transport.

**Figure 8:**
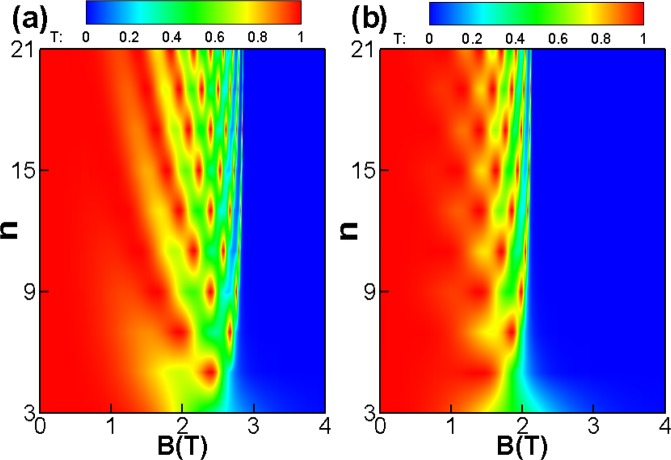
The (*B*, *n*) dependence of transmission probability through the periodic delta-function-shaped magnetic barriers for (**a**) *γ* = 0 and (**b**) *γ* = *π*/6. The incident angle *φ* = 0, *E*_*F*_ = 78 *meV*, *D* = 26 *nm*, and *L* = 26 *nm*.

Figure 8 shows the contour plot of the transmission probability *T*(*B*,*n*) for the case of a periodic delta-function-shaped magnetic barriers. The incident angle is *γ* = 0 for Fig. 8(a) and *γ* = *π*/6 for Fig. 8(b), respectively. One can see clearly that the tunneling is totally forbidden at the cut-off magnetic field *B* = *E*_*F*_*l*_*B*_cos*γ*(1 − sin*φ*)*B*_0_/*DE*_0_. The cut-off magnetic field would not decrease as superlattice number *n* increases. On the contrast, the critical magnetic field becomes weaker as increasing superlattice number *n* for the case of the square-shaped magnetic barriers [see Fig. 4(a,b)].

### The magnetoresistance

The magnetoresistance ratio is defined as *MR* = (*G*_*P*_ − *G*_*AP*_)/*G*_*AP*_, where the *G*_*P*_(*G*_*AP*_) denotes the conductance of the parallel (antiparallel) configuration. In terms of the Landauer-Büttiker formalism, we can obtained the ballistic conductance, $G={G}_{0}{\int }_{-\infty }^{\infty }{\int }_{-{k}_{F}}^{{k}_{F}}{\int }_{-{k}_{F}}^{{k}_{F}}TdEd{k}_{y}d{k}_{z}$, where *G*_0_ = *e*^2^*L*_*y*_*L*_*z*_/(*πh*) is the conductance unit, *L*_*y*_(*L*_*z*_) is the length in the *y* (*z*) direction.

**Figure 9:**
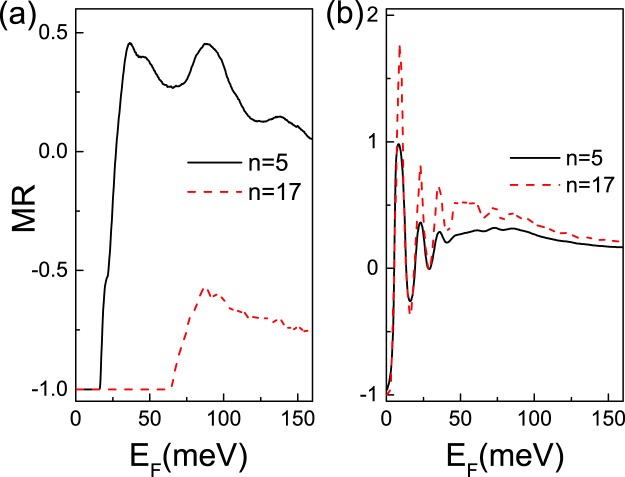
The magnetoresistance ratio MR as a function of the incident energy *E*_*F*_ for a (**a**) square-shaped magnetic barrier (**b**) delta-function-shaped magnetic barrier. Here *D* = 26 nm, *L* = 78 nm, *B* = 1 T.

The magnetoresistance ratio is shown as a function of *E*_*F*_ for different superlattice numbers *n* (Fig. 9). The MR for square-shaped barrier is smaller than that for delta-function-shaped barrier (see Fig. 9(a,b)). All the magnetoresistance ratio MR becomes negative with the superlattice number increasing. For the parallel square-shaped configuration, with the increase of the superlattice number, the vector potential *A*_*y*_ in the outgoing region increases. When the vector potential *A*_*y*_ is past a critical value, the outgoing wave becomes evanescent spatially, thus *G*_*P*_ decreases. While for the antiparallel square-shaped configuration, the transmission is independent of the superlattice number. Therefore, *MR* changes from the positive to negative with the increase in the superlattice number. In the case of delta-function-shaped barrier, there is significant oscillation in MR(see Fig. 9(b)). The increase in superlattice number reduces the differences in parallel square-shaped configuration and antiparallel square-shaped configuration, thus leads to MR increase.

## Conclusion

In summary, we investigate theoretically the effect of periodic magnetic barriers on transport for a Weyl semimetal. The transmission has an interesting momentum and spin filtering feature which can be tuned by the number of the magnetic barriers. The transmission probability *T* becomes zero beyond a certain magnetic field value. The critical magnetic field is proportional to the number of the superlattice for the tunneling through periodic square-shaped magnetic barriers, but independent of the number of the superlattice for the tunneling through periodic delta-function-shaped magnetic barriers. The constructive interference of the backscattering by different periodic magnetic barriers results in the formation of superlattice minigaps and switches off the transport. The tunneling magnetoresistance depends on the number of the magnetic barriers. These behaviors offer us an efficient way to control the transport and construct Weyl semimetal-based electronic devices.
